# CpG island hypermethylation-associated silencing of microRNAs promotes human endometrial cancer

**DOI:** 10.1186/1475-2867-13-44

**Published:** 2013-05-16

**Authors:** Bi-Lan Li, Wen Lu, Cong Lu, Jun-jie Qu, Ting-ting Yang, Qin Yan, Xiao-ping Wan

**Affiliations:** 1 Department of Obstetrics and Gynecology, Shanghai First People’s Hospital Affiliated to Shanghai Jiao Tong University School of Medicine, 650 New Songjiang Road, Shanghai 201620, China

**Keywords:** DNA methylation, Histone modification, Microrna, Endometrial cancer, DICER1, Metastasis, Epithelial mesenchymal transition

## Abstract

**Background:**

Endometrial cancer (EC) is the most common gynecologic malignancy, but the molecular events involved in the development and progression of EC remain unclear. This study aimed to explore epigenetic modification of genes and miRNAs involved in EC development.

**Methods:**

Ishikawa and AN3CA cells were treated with 5’-Aza-2-deoxycytidine or histone deacetylase inhibitor. The expression of miRNAs and related genes were detected by PCR and Western blot. Promoter methylation was detected by bisulfite specific PCR sequencing. The proliferation, colony formation, cell cycle progression, migration and invasion of EC cells were evaluated by MTT, soft agar assay, flow cytometry, wound healing and invasion assay, respectively.

**Results:**

Aberrant expression of miRNAs including miR-200b, miR-130a/b, miR-625 and miR-222 was associated with tumorigenesis and metastasis in endometrial cancer. Silencing of miR-130b induced E-cadherin expression, while ectopic expression of miR-130b and knockdown of DICER1 increased the expression of Vimentin, zeb2, N-cadherin, Twist and Snail in EC cells. Furthermore, 5’-Aza-2-deoxycytidine and Histone deacetylase (HDAC) inhibitor inhibited the proliferation, colony formation, migration and invasion of EC cells, accompanied by reduced MMP secretion.

**Conclusions:**

Our study provides the first description of epigenetic modification of epithelial mesenchymal transition associated genes and miRNAs in EC cells, which are extensively involved in the regulation of gene expression and subsequent accumulation of malignant features of EC cells.

## Background

Endometrial cancers are one of the most common gynecological cancers in the United States, with over 35,000 women diagnosed each year. Endometrial endometrioid carcinomas (EECs) represent 80-85% of all endometrial cancers [1]. When diagnosed at an early stage, the prognosis for EC has improved over recent years [2]. However, for patients diagnosed with late-stage disease they have an overall poor prognosis [3]. Therefore, there is urgent need to further understand the molecular mechanism underlying the development and progression of EEC.

Recent evidence has suggested that epigenetic mechanisms contribute to the development, progression and metastasis of cancer including endometrial cancer [4,5]. These epigenetic changes occur apart from primary genomic sequences and include DNA methylation, histone modifications, and miRNA expression [6]. In human neoplasias, CpG island hypermethylation is associated with transcriptional silencing of tumor suppressor genes including genes that encode miRNAs, which are produced by DICER1, a cytoplasmic RNase III enzyme [7]. miR-130b hypermethylation was found in ovarian cancer tissues as well as in drug-resistant cell lines [8].

The irreversible loss of E-cadherin expression emerges as a critical step driving epithelial mesenchymal transition (EMT) in various human cancers. The loss of E-cadherin expression increases tumor invasiveness in vitro and in vivo and also increases the resistance of cancer cells to chemotherapeutic agents. Recent reports have implicated a crucial role for the miR-200 family in the regulation of E-cadherin transcriptional repressors zinc finger E-box binding homeobox 1 (ZEB1) and zinc finger E-box binding homeobox 2 (ZEB2) [9]. In addition, the downregulation of DICER1 has been associated with the miR-200 family-EMT pathway and tumor metastasis, which indicates poorer prognosis [10].

Here we presented for the first time a comprehensive analysis of miR-130 family and DICER1 expression in endometrial cancer tissues, compared with normal endometrium. In addition, with EC cells as experimental model we explored the mechanism and functional consequences of dysregulation of some miRNAs, whose expression was linked to aberrant DNA methylation and histone modification and regulated the growth and invasion of EC cells.

## Materials and Methods

### Cell culture and treatment

The human endometrial cell lines Ishikawa and AN3CA were obtained from the Chinese Academy of Sciences Committee Type Culture Collection cell bank. The cells were grown in Dulbecco’s modified Eagle’s medium (DMEM)/F12 (11030; Gibco, Auckland, New Zealand) supplemented with 10% fetal bovine serum (FBS) (16000–44; Gibco, Carlsbad, CA), 100 u/mL penicillin, and 100 μg/mL streptomycin in a humidified atmosphere of 5% CO_2_/95% air at 37°C. The cells were treated with 10 μM 5’-Aza-2-deoxycytidine (Sigma) or 10 μM HDAC inhibitor:Trichostatin A (TSA) (Sigma).

### Cell transfection

Cells were washed with PBS and transiently transfected with 100 nM pre-miR-130b or anti-miR-130b with their corresponding negative controls (miR negative control #1 or anti-miR negative control#1; Applied Biosystems; Foster City, CA, USA) in Opti-MEM (Invitrogen, Carlsbad, CA, USA) using siPORT NeoFX transfection agent (Applied Biosystems; Foster City, CA,USA) following the manufacturer’s protocol. Medium was replaced 8 h later. small interfering RNA (siRNA) expression vectors targeting DICER1 were transiently transfected into AN3CA and Ishikawa cells using lipofectamine 2000 following the manufacturer’s instructions.

### Quantitative real-time PCR

Fresh-frozen EEC tissue samples and normal endometrial samples were obtained from patients at the Obstetrics and Gynecology Department of Shanghai First People’s Hospital, affiliated to Shanghai Jiao Tong University School of Medicine. Following excision, tissue samples were immediately snap-frozen in liquid nitrogen and stored at −80°C until RNA extraction. Total RNA was extracted from the tissues or cells using TRIzol RNA Isolation Reagents (Invirogen). The cDNA was generated using Prime Script RT reagent Kit (TaKaRa, Dalian, China). A 50 μL PCR amplification of single-strand cDNA was performed with 40 cycles of denaturation (94°C) for 60 s, annealing (55°C) for 30 s, and elongation (72°C) for 30 s using PerfectShot Ex Taq (Loading Dye Mix) (DRR05TA; Takara, Dalian, China). The primer sequences were as follows: DICER1 Forward TTCCAAGTCGGTTGATACTGG, Reverse TGTTGATTGTGACTCGTGGAC; Zeb2(sip1) Forward AGCAGGTAATCGCAAGTTCAA, Reverse AGTTTGGGCACTCGTAAGGTT; Vimentin Forward ATGCGTGAAATGGAAGAGAACT, Reverse CTCAGGTTCAGGGAGGAAAAGT; E-cadherin Forward GCTACTHHAACAGGGACACTTC, Reverse TTCTGCTGTGAAGGGAGATGTA; β-actin Forward CAG AGC AAG AGA GGC ATC C, Reverse CTG GGG TGT TGA AGG TCT C

Real-time quantitative PCR of miRNAs was performed using TaqMan assay (Applied Biosystems, Foster City, USA). The relative fold change was calculated based on the differences in Ct values (ΔCt) between fold change = 2ΔΔ-Ct. Three biological and technical replicates were done for each sample. All values were expressed as mean ± standard deviation.

### Bisulfite specific PCR sequencing

The miRNA sequences were analyzed by using miRBase (http://microrna.sanger.ac.uk/) and the University of California at Santa Cruz Human Genome Browser (http://genome.cse.ucsc.edu). The CpG Island Searcher Program was used to determine which miRNAs were embedded in CpG islands. Genomic DNA was isolated from cells using Trizol (Invitrogen), and 500 ng grnomic DNA was bisulfite modified using the EZ DNA Methylation-Gold Kit (Zymo Research, Irvine, CA) according to the manufacturer’s protocols. Two procedures were used. First, methylation status was analyzed by bisulfite modified DNA sequencing of the corresponding CpG islands. Six independent clones were analyzed. The PCR was performed using a Rotor-Gene 3000 with 45 cycles of denaturation (95°C) for 30 s and annealing (53°C) for 60 s, and a final extension at 72°C for 4 min. PCR products were subcloned into T-easy vector for sequencing.

### Western blot analysis

Cells were washed with ice-cold PBS and lysed in ice-cold RIPA (Beyotime) on ice for 30 min. Total protein was measured using Bio-Rad protein assay reagent according to the manufacturer’s protocol. Protein (60 μg) was seperated by 10% PAGE gels and transfered to Polyvinylidene Fluoride (PVDF) membranes. After washing with tris-buffered saline, the membranes were blocked with 5% bovine serum albumin (BSA)/phosphate buffered saline (PBS) for 1 h, incubated at 4°C overnight with primary antibodies against DICER1 (1:1000 dilution, ab39742, Abcam, UK), E-CADHERIN (1:1000 dilution, CST, USA), VIMENTIN (1:1000 dilution, CST,USA), ZEB2 (1:1000 dilution,Epitomics), Twist1 (1:1000 dilution, Epitomics), Snail (1:1000 dilution, Epitomics), N-cadherin (1:1000 dilution, CST, USA) and β-actin (1:1000 dilution, 4697s, CST, USA). The membranes were washed three times with PBS and then incubated with peroxidase-linked secondary antibody (1:10 000) for 1 h at room temperature. The signals were developed using an ECL kit (Pierce), scanned, and analyzed with Total Lab software. The relative expression of target proteins was presented as the ratio to β-actin.

### Cell invasion assay

Cell invasion was assessed by using a BD BioCoat Matrigel Invasion Chamber (BD Biosciences) according to the manufacturer’s instructions. Cells (5 × 10^4^ cells/ml) were loaded into chamber inserts containing an 8 μm pore size membrane with a thin layer matrigel matrix. Cells migrating to the lower surface of the membrane during 48 h were fixed with 100% methanol. The membranes were then stained with hematoxylin, scanned, and analyzed with an Aperio Scanscope System (Aperio Technologies, Vista, CA).

### Flow cytometry of cell cycle

Cells were fixed with 70% ethanol for 72 h and stained with 25 μg/mL propidium iodide (PI, Boster) in fluorescence-activated cell sorting buffer (PBS containing 0.1% bovine serum albumin, 0.05% of Triton X-100, and 50 μg /mL RNaseA) for 30 min at room temperature in the dark., the cells were analyzed by flow cytometry using a Becton Dickinson FACScan. Experiments were performed in triplicate in three independent experiments.

### Proliferation assay

Cells were cultured in phenolred-free medium containing 5% charcoal-stripped FBS (HyClone Laboratories; Logan, Utah, USA). Cell proliferation was analyzed every 24 h via colorimetric assay with 3-(4, 5-dimethylthiazol-2-yl)-2, 5-diphenyltetrazolium bromide (MTT) (SIGMA; St. Louis, MO, USA). Absorbance at 490 nm was evaluated by a Spectra Max 190 microplate reader (Molecular Devices; Sunnyvale, CA, USA). Experiments were performed in triplicate in three independent experiments.

### Soft agar colony assay

Cells were seeded in 0.3% top agar in growth medium over a layer of 0.6% agar in a 6-well plate at a density of 1 × 10^4^ cells/well. After 3 weeks of incubation, colonies with more than 50 cells were counted and photographed with an inverted microscope. The assay was performed at least three times in triplicate.

### Statistical analysis

Each experiment was performed as least three times, and data are shown as the mean ± SD where applicable, and differences were evaluated using one-way ANOVA for 3-group comparisons and t tests for 2-group comparisons. All statistical analyses were performed using SPSS 13.0 software package. *P < 0.05* was considered to be statistically significant.

## Results

### Methylation status of miRNAs in human endometrial cancer cells treated with demethylation agents and histone deacetylase inhibitor

**Figure 1:**
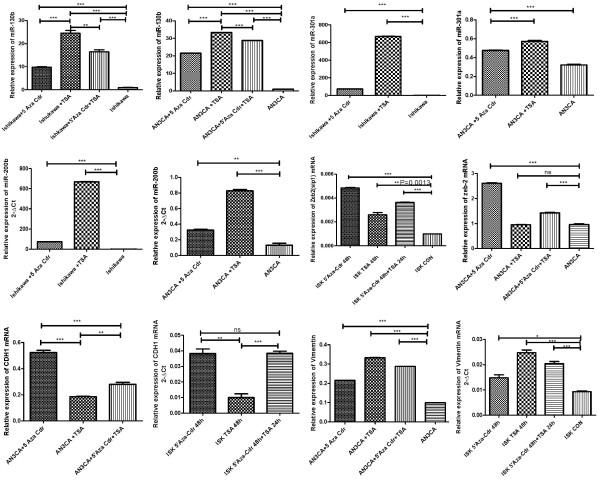
**Epigenetic silencing of miRNAs and EMT-related genes by CpG hypermethylation, and histone modification in endometrial cancer cells.** qRT-PCR analysis of miR-130b, miR-301a, and miR-200b expression as well as DICER1, zeb2, CDH1, and vimentin mRNA expression levels in Ishikawa and AN3CA cells treated with 10 μM 5'-aza-CdR and 10 μM TSA. Data were shown as fold change. * *P* < 0.05, ** *P* < 0.01, compared to DMSO treatment.

miR-130a/b, miR-200b, and miR-625 contain several CpG sites in their upstream regulatory sequences. We assessed the methylation status of these CpG islands in both EECs and normal endometrium by bisulfite-specific PCR sequencing. We detected hypomethylation of miR-130b in EECs. After treatment with demethylation agents for 72 h, the expression of miR-130b increased 36.8-fold in Ishikawa cells and 29.6-fold in AN3CA cells (*P* < 0.01) (Figure 1). Furthermore, following treatment with HDAC inhibitor, the expression of miR-130b was upregulated 21.2-fold in Ishikawa cells and 23.3-fold in AN3CA cells. Surprisingly, the methylation level was found to be mildly decreased, suggesting a role for HDAC inhibition in modulating the DNA methylation status. The EMT-related genes, miR-200b, miR-130a, zeb2, and E-cadherin were also upregulated by demethylating agents. Conversely, DICER1 and vimentin were downregulated by these agents (Figure 1).

**Figure 2:**
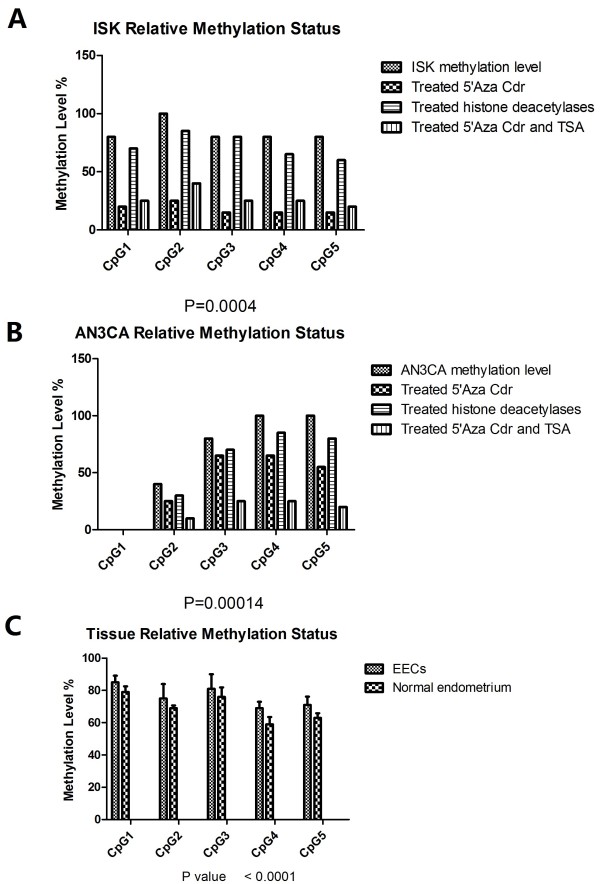
**Bisulfite specific PCR sequencing to assess CpG islands of miR130b.** Box plots showed the mean ± SD. **A**. Relative methylation levels in Ishikawa cells treated with 5’-Aza-Cdr (10 μM) for 48 h, TSA (10 μM) for 48 h, or 5’-Aza-Cdr for 48 h plus TSA for 24 h. **B**. Relative methylation levels in AN3CA cells treated with 5’-Aza-Cdr (10 μM) for 48 h, TSA (10 μM) for 48 h, or 5’-Aza-Cdr for 48 h plus TSA for 24 h. (**C**) Different levels of methylation between normal endometrium and EECs.

We further examined whether miR-130b expression was regulated by CpG methylation. Compared to normal endometrium tissue, EECs displayed significantly lower levels of methylation, and the level of miR-130b was negatively correlated with CpG methylation (Figure 2).

To explore the mechanisms underlying the upregulation of miRNAs in endometrial cancers, we examined the methylation status of miR-130a, miR-130b, miR-625 and miR-200b by bisulfite-specific PCR sequencing (Table 1). These miRNAs were epigenetically regulated through the associated CpG islands, and the methylation levels were closely linked with the expression of these miRNAs (Figure 2). We also performed bisulfite-specific PCR sequencing for DICER1 in Ishikawa cells and found that the methylation status was not related with the expression of DICER1 (data not shown).

**Table 1:** Bisulfite specific PCR sequencing of miRNAs

**GENE**	**Cell type**	**Bisulfite specific PCR sequencing**													
		**CpG-1**		**CpG-2**		**CpG-3**		**CpG-4**		**CpG-5**		**CpG-6**		**CpG-7**	
		**gene locus**	**probability**	**gene locus**	**probability**	**gene locus**	**probability**	**gene locus**	**probability**	**gene locus**	**probability**	**gene locus**	**probability**	**gene locus**	**probability**
miR-130a	AN3CA	26	100%	120	100%	227	100%	276	0	/	/	/	/	/	/
	Ishikawa		80%		100%		80%		0		/		/		/
miR-130b	AN3CA	33	0	46	40%	119	80%	151	100%	183	100%	/	/	/	/
	Ishikawa		80%		100%		80%		80%		80%		/		/
miR-625	AN3CA	24	100%	17	100%	53	100%	68	100%	90	100%	/	/	/	/
	Ishikawa		100%		80%		100%		60%		100%		/		/
miR-200b	AN3CA	26	29%	55	100%	77	86%	81	86%	88	86%	99	86%	107	86%
	Ishikawa		40%		100%		100%		80%		80%		80%		60%
miR-222	AN3CA	122	100%	/	/	/	/	/	/	/	/	/	/	/	/
	Ishikawa		100%		/		/		/		/		/		/

### miR130b and DICER1 regulate EMT realted genes

**Figure 3:**
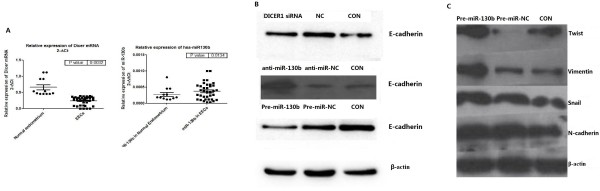
**miR130b and DICER1 regulate EMT realted genes. A**. The expression and correlation of miR-130b and DICER1 in clinical samples. The scatter plots indicated an inverse correlation between miR-130b and DICER1 mRNA expression, as determined by real-time PCR. **B**, **C**. Western blots showing E-cadherin, N-cadherin, Twist1, Snail, Zeb2 and vimentin expression levels in AN3CA cells after transfection with DICER1 siRNA, anti-miR130b or pre-miR130b.

We compared the expression of miR-130b and DICER1 between endometrial cancers and normal endometrium. qRT-PCR analysis indicated that miR-130b was lower in normal endometrium than in endometrial cancer while DICER1 was higher in normal endometrium than in endometrial cancer (Figure 3A). These data indicated that miR-130b was inversely correlated with DICER1 expression at the mRNA level.

To understand the role of miR-130b and DICER1 in the regulation of EMT, we manipulated the expression of miR-130b and DICER1 in EC cells and examined the effects on the expression of EMT-related genes such as E-cadherin, Twist, Snail, N-cadherin, zeb2 and vimentin. Ishikawa and AN3CA cells were transiently transfected with anti-miR-130b inhibitor and anti-negative control (anti-NC), along with DICER1 siRNA and siRNA negative control. The results showed that transfection of pre-miR-130b upregulated vimentin, N-cadherin, Twist, zeb2 and Snail expression, but downregulated E-cadherin expression. In contrast, transfection of DICER1 siRNA downregulated E-cadherin expression (Figure 3B, C). These results suggest that miR-130b and DICER1 have opposite effects on the regulation of EMT.

### 5’-Aza-2-deoxycytidine and HDAC inhibitor regulate biological behaviors of endometrial cancer cells

**Figure 4:**
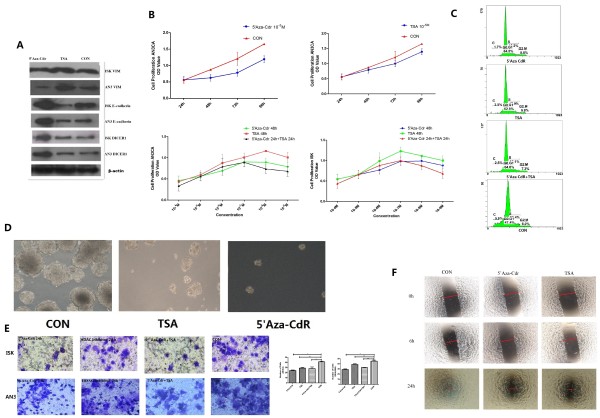
**5’-Aza-2-deoxycytidine and HDAC inhibitor regulate biological behaviors of EC cells*. *A**. Western blots showing DICER1, E-cadherin and vimentin expression levels in Ishikawa and AN3CA cells treated with 10 μM 5'-aza-CdR and 10 μM TSA. **B**. The proliferation of AN3CA cells treated as indicated. **C**. The cell cycle prolifes of AN3CA cells treated as indicated. **D**. The colony formation of AN3CA cells treated as indicated. **E**. Transwell invasion assay of AN3CA and Ishikawa cells treated as indicated. Representative images of AN3CA and Ishikawa cells were shown (magnification × 200). Statistical analysis of the number of invaded Ishikawa and AN3CA cells. **F**. *in vitro* wound healing assay of AN3CA cells. Photographs were taken at 0, 6 and 24 h after the wound was made.

After incubation with 5’-Aza-2-deoxycytidine and HDAC inhibitor for 48 h, the expression of DICER1, E-cadherin and Vimentin were analyzed by Western blot. The expression of DICER1 and E-cadherin protein were up-regulated significantly in the cells treated with 5’-Aza-2-deoxycytidine or HDAC inhibitor compared with the control, while the expression of Vimentin was down-regulated significantly in the cells treated with 5’-Aza-2-deoxycytidine (Figure 4A).

The proliferation assay showed that 5’-Aza-2-deoxycytidine and HDAC inhibitor inhibited the growth of EC cells in a time-dependent manner (Figure 4B). Flow cytometry showed that in AN3CA and Ishikawa cells demethylation agents caused an increase of cells in G0/G1 phase and a reduction of cells in S phase (Figure 4C and Tables 2 and 3). We went on to investigate whether 5’-Aza-2-deoxycytidine and HDAC inhibitor could inhibit anchorage-independent growth, a hallmark of oncogenic transformation. The soft agar assay showed that the colony formation of AN3CA cells in soft agar was significantly inhibited by treatment with 5’-Aza-2-deoxycytidine or TSA (Figure 4D).

**Table 2:** The cell cycle prolifes of AN3CA cells

**Cell cycle fraction**	**Blank**	**10 μM HDAC inhibitor for 48 h**	**10 μM 5’-Aza-2-deoxycytidine for 48 h**	**10 μM 5’-Aza-2-deoxycytidine for 24 h 10 μM + HDAC inhibitor for 24 h**
G0-G1	40.3	63.4	64.2	65.2
S	20.7	17.31	16.64	16.01

**Table 3:** The cell cycle prolifes of Ishikawa cells

**Cell cycle fraction**	**Blank**	**10 μM HDAC inhibitor for 48 h**	**10 μM 5’-Aza-2-deoxycytidine for 48 h**	**10μM 5’-Aza-2-deoxycytidine for 24 h 10μM + HDAC inhibitor for 24 h**
G0-G1	40.3	62.4	63.8	66.4
S	30.7	15.31	14.33	13.92

Using transwell chambers precoated with Matrigel, we examined the effect of demethylation agents and HDAC inhibitor on the invasion of EC cells. AN3CA and Ishikawa cells treated with demethylation agents and HDAC inhibitor showed significantly decreased invasiveness compared with control and untreated cells (Figure 4E). In contrast, the controls showed no effect. Similar results were obtained in wound-healing assays with aggressive AN3CA cells (Figure 4F).

Taken together, these results demonstrate that DNA hypermethylation and histone deacetylation cooperate to regulate the growth and invasion of endometrial cancer cells.

### 5’-Aza-2-deoxycytidine and HDAC inhibitor inhibit the secretion of Matrix metalloproteinase 2 (MMP-2) and Matrix metalloproteinase 9(MMP-9) in endometrial cancer cells

**Figure 5:**
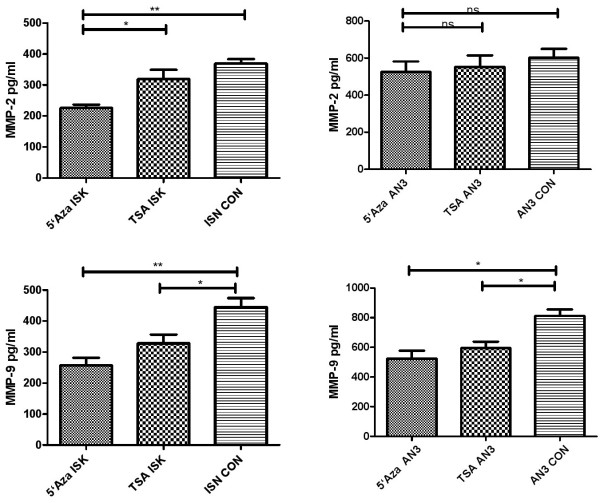
**5’-Aza-2-deoxycytidine and HDAC inhibitor regulate the secretion of MMPs in EC cells*. ***MMP-2 and MMP-9 secretion was decreased significantly in cells treated with 5’Aza-Cdr (10^–5^ M) or TSA (10^–5^ M) The data are expressed as mean concentrations with SD. * P < 0.05, ** P < 0.01.

To understand the mechanims by which DNA hypermethylation and histone deacetylation regulate the invasion of endometrial cancer cells, we focused on MMPs, which are positive regulators of cancer invasion. Using an ELISA kit, we detected MMP-2 and MMP-9 levels in cultured supernatants from AN3CA and Ishikawa cells treated with 5’Aza-Cdr (10^–5^ M) or TSA (10^–5^ M). The results showed that the secretion of MMP-2 and MMP-9 was inhibited by 5’Aza-Cdr or TSA (Figure 5). These data suggest that DNA hypermethylation and histone deacetylation regulate the invasion of endometrial cancer cells via the regulation of MMPs.

## Discussion

Although endometrial cancer consists of multiple tumor types, EEC is the most common. DNA methylation, histone modifications and miRNA regulation have emerged as key elements regulating tumorigenesis and cancer progression. In this present study we found that aberrant expression of miRNAs including miR-200b, miR130a/b, miR-625 and miR-222 was associated with tumorigenesis and metastasis in endometrial cancer. We analyzed the microRNA signatures associated with EC invasion and determined their relationships with EMT markers including E-cadherin, vimentin, and miR-200 family. The loss of epithelial markers such as E-cadherin and the acquisition of a mesenchymal phenotype such as Vimentin were accompanied by the changes in the levels of miRNAs.

We found dramatic differential expression of miR-130b and the level of its CpG methylation associated with EMT-related genes in endometrial cancer cells treated with 5’-Aza-Cdr or TSA, compared to untreated cells. Therefore, histone acetylation and DNA methylation may form a complex framework for epigenetic control of the development of EC. It has recently become apparent that DNA methylation and histone modification may be dependent on each other, and their cross-talk is most likely mediated by biochemical interactions between SET domain of histone methyltransferases and DNA methyltransferases [11]. Here we showed that HDAC inhibitor activated gene expression through the changes in the histone methylation status, which is coordinated with DNA methylation.

Notably, we found that 5’-Aza-CdR reversed the hypermethylation of miR-130b promoter and inhibited the maglinant behaviors of EC cells. These findings demonstrate that particular DNA methylation of miRNAs is associated with aggressive tumor behaviors and suggest that CpG island hypermethylation-mediated silencing of cancer-related miRNAs contributes to human tumorigenesis. An important issue of our study presented here is the mechanism by which demethylating agents and HDAC inhibitors cause dysregulation of miR-130b expression. One hypothesis is that HDAC inhibitor induces the increases in chromatin acetylation, leading to the expression of a factor that represses miRNA synthesis [6]. Alternatively, HDAC inhibitors may disrupt the repressive transcriptional complex that binds to miR-130b regulatory elements, leading to miR-130b up-regulation and consequent inhibition of E-cadherin expression.

Our results showed that demethylation agents and HDAC inhibitor inhibited the proliferation and colony formation of EC cells, as well as the migration and invasion of EC cells. EMT is a crucial event in tumor progression, and it is associated with dysregulation of DICER1, E-cadherin and miR-200 family, and upregulation of vimentin, N-cadherin, Twist1, Snail and Zeb2. In this study we showed that specific miRNAs, particularly miR-130a/b and miR-200 family, were crucially involved in gene expression during EMT and the subsequent accumulation of malignant features. In particular, silencing of miR-130b induced E-cadherin expression to inhibit EMT process, while ectopic expression of miR-130b and knockdown of DICER1 increased the expression of Vmentin, zeb2, N-cadherin, Twist and Snail to promote EMT process. A large body of evidence suggests that the multigene regulatory capacity of miRNAs is dysregulated and exploited in cancer and miRNA signatures have been associated with clinical outcomes of a variety of cancers including endometrial cancer [12-15]. Recently, miR-152 was identified as a tumor suppressor microRNA that was silenced by DNA hypermethylation in endometrial cancer [16]. Consistent with the epigenetic regulation of miRNAs we further showed that demethylation agent or HDAC inhibitor inhibited the secretion of MMP-2 and MMP-9 in EC cells, which further proves that epigenetic regulation of miRNAs play a role in the regulation of EMT and tumor metastasis of EC.

In addition to conventional mechanisms of gene inactivation, epigenetic changes of specific miRNAs, including gain and loss of DNA methylation and altered histone modifications, are considered hallmarks of human cancer. Reversal of DNA methylation and histone modifications could potentially be therapeutic, as epigenetic modifications result in stable, heritable changes in gene expression without altering genetic sequences or gene function. Very recently, demethylating agent 5’-aza-CdR was shown to synergize with progesterone therapy to inhibit EC cell growth and invasion [17].

## Conclusions

To our knowledge, in this study we provide the first description of epigenetic modification of EMT associated genes and miRNAs in EC cells. We show that specific miRNAs along with DNA methylation and histone modifications are extensively involved in the regulation of gene expression and subsequent accumulation of malignant features of EC cells. These findings suggest that miRNAs combined with demethylation agents and histone modification agents could be potentially utilized for endometrial cancer therapy.

## Competing interests

The authors declare that they have no competing interests.

## Authors’ contributions

BL, WL, CL, JQ performed most of the experiments. BL and XW designed the study. TY and QY performed statistical analysis. XW supervised the study and the preparation of the manuscript. All authors have read and approved the final version of the manuscript.

## References

[B1] Boll D Verhoeven RH van der Aa MA Incidence and survival trends of uncommon corpus uteri malignancies in the Netherlands, 1989–2008 Int J Gynecol Cancer 2012 22 599 606 10.1097/IGC.0b013e318244cedc 22398706

[B2] Lajer H Elnegaard S Christensen RD Ortoft G Schledermann DE Mogensen O Survival after stage IA endometrial cancer; can follow-up be altered? A prospective nationwide Danish survey Acta Obstet Gynecol Scand 2012 91 976 982 10.1111/j.1600-0412.2012.01438.x 22548255

[B3] Azueta A Gatius S Matias-Guiu X Endometrioid carcinoma of the endometrium: pathologic and molecular features Semin Diagn Pathol 2010 27 226 240 10.1053/j.semdp.2010.09.001 21309258

[B4] Feller L Kramer B Lemmer J Pathobiology of cancer metastasis: a short account Cancer Cell Int 2012 12 24 10.1186/1475-2867-12-24 22676510 PMC3407798

[B5] Falck E Karlsson S Carlsson J Helenius G Karlsson M Klinga-Levan K Loss of glutathione peroxidase 3 expression is correlated with epigenetic mechanisms in endometrial adenocarcinoma Cancer Cell Int 2010 10 46 10.1186/1475-2867-10-46 21106063 PMC3014921

[B6] Allis CD Berger SL Cote J New nomenclature for chromatin-modifying enzymes Cell 2007 131 633 636 10.1016/j.cell.2007.10.039 18022353

[B7] Hammond SM Dicing and slicing: the core machinery of the RNA interference pathway FEBS Lett 2005 579 5822 5829 10.1016/j.febslet.2005.08.079 16214139

[B8] Creasman W Revised FIGO staging for carcinoma of the endometrium Int J Gynaecol Obstet 2009 105 109 10.1016/j.ijgo.2009.02.010 19345353

[B9] Kumar MS Lu J Mercer KL Impaired microRNA processing enhances cellular transformation and tumorigenesis Nat Genet 2007 39 673 677 10.1038/ng2003 17401365

[B10] Karube Y Tanaka H Osada H Reduced expression of DICER1 associated with poor prognosis in lung cancer patients Cancer Sci 2005 96 111 115 10.1111/j.1349-7006.2005.00015.x 15723655 PMC11158408

[B11] Cedar H Bergman Y Linking DNA methylation and histone modification: patterns and paradigms Nat Rev Genet 2009 10 295 304 19308066 10.1038/nrg2540

[B12] Calin GA Croce CM MicroRNA signatures in human cancers Nat Rev Cancer 2006 6 857 866 10.1038/nrc1997 17060945

[B13] Golestaneh AF Atashi A Langroudi L miRNAs expressed differently in cancer stem cells and cancer cells of human gastric cancer cell line MKN-45 Cell Biochem Funct 2012 30 411 418 10.1002/cbf.2815 22374783

[B14] Bae J Won M Kim DY Identification of differentially expressed microRNAs in endometrial cancer cells after progesterone treatment Int J Gynecol Cancer 2012 22 561 565 10.1097/IGC.0b013e31824927db 22543862

[B15] Torres A Torres K Paszkowski T Highly increased maspin expression corresponds with up-regulation of miR-21 in endometrial cancer: a preliminary report Int J Gynecol Cancer 2012 22 951 959 10.1097/IGC.0b013e3182540160 21330826

[B16] Tsuruta T Kozaki K Uesugi A miR-152 is a tumor suppressor microRNA that is silenced by DNA hypermethylation in endometrial cancer Cancer Res 2011 71 6450 6462 10.1158/0008-5472.CAN-11-0364 21868754

[B17] Hu Q Yu L Chen R 5-aza-2'-deoxycytidine improves the sensitivity of endometrial cancer cells to progesterone therapy Int J Gynecol Cancer 2011 21 8 14 10.1097/IGC.0b013e318200050e 22683940

